# Editorial: Physiological, anatomical and sport performance adaptations to concentrated training periods in athletes

**DOI:** 10.3389/fspor.2024.1491863

**Published:** 2024-10-02

**Authors:** Andrew S. Perrotta, Jared R. Fletcher

**Affiliations:** ^1^ Department of Kinesiology, Faculty of Human Kinetics, University of Windsor, Windsor, ON, Canada; ^2^ Department of Kinesiology, Centre for Human Performance and Health, Windsor, ON, Canada; ^3^ Department of Health and Physical Education, Mount Royal University, Calgary, AB, Canada

**Keywords:** sport science, athlete monitoring, sport medicine, human performance, exercise physiology

**Editorial on the Research Topic**
Physiological, anatomical and sport performance adaptations to concentrated training periods in athletes

## Introduction

Understanding the dose-response to exercise has become the central responsibility of sports scientists. The complex physiology of an athlete requires practitioners to develop a comprehensive “fingerprint” that is unique to the athlete's personal response to exercise, training methodologies, and periodization. The dose-response to exercise as described by (1) typically involves an initial, brief period of fatigue, that can develop into long-term improvement in biological function if appropriate rest and recovery are provided. This model is largely based on Selye's explanation of the fundamental reaction to the experience of whole-body stress over a continuous period, whereby positive adaptations or exhaustion can occur depending on the sustained stress experienced (2). The “dose” of exercise can be quantified into a training load metric using either wearable technology (3) or a subjective assessment of perceived exertion (4, 5), which can be compared to the resulting physiological “response” (6, 7). When examining them together, practitioners can make informed decisions when prescribing impending training sessions depending on the “response” to the “dose” of exercise. For example, wearable devices that include GPS technology can accurately measure the speed and distance of a training session (i.e., the “dose”). Combined with information from a simple heart rate monitor (i.e., the “response”), coaches and practitioners can periodically evaluate the relationship between heart rate and speed in the field as an indirect marker of exercise economy/efficiency and may indirectly serve to indicate states of transient or long-lasting fatigue (8, 9). Analyzing the dose-response relationship to exercise can be valuable to coaches, athletes and practitioners. In a 2012 survey of coaches and sport science support staff, Taylor et al. (10), found that 91%of respondents used some form of a training monitoring system. In this survey, 70%of these respondents indicated that the focus was on “load quantification” and monitoring fatigue or recovery to “prevent overtraining, reduce injuries, monitor the effectiveness of the training programs and ensure maintenance of performance” (10).

This Research Topic of Frontiers in *Physiology and sports and active living*, contains four manuscripts with the primary purpose of analyzing the dose-response relationship to exercise to improve athletic development. Three original articles (Perrotta et al.; Liu et al.; Rice et al.) and one systematic meta-analysis (Yogev et al.) meet the editorial criteria.

Perrotta et al. aimed to examine the dose-response from performing soccer-specific activities designed to improve the technical and tactical performance of players in addition to their physiological functioning during a 4-week pre-season period in female collegiate soccer players. They found that on-field training alone can promote positive and significant adaptations in body composition, resting cardiovascular function, and indices of athletic performance measures. The magnitudes of these adaptations were associated with both internal and external measures of accumulated exercise stress. Preseason training often involves a strong emphasis on accumulated exercise stress to enhance player fitness levels before the commencement of the regular season. The findings from this study emphasize that a collaborative approach between coaches and practitioners is crucial when designing on-field training sessions that address both technical/tactical aspects, as the resulting physiological stress may also be conducive to enhancing physical performance. This outcome may eliminate the need for additional resistance training sessions thereby promoting the recovery and overall development of athletes before the regular season begins.

Rice et al. addressed a gap in the literature by examining off-ice predictors of on-ice performance in youth ice hockey. Their work has important implications because athlete developmental pathways generally begin as early as 6 years of age. The authors identified 10 key off-ice determinants of on-ice performance including sprinting speed and acceleration, agility, anaerobic power, aerobic power and lower leg strength (assessed by a one-leg squat measurement). Importantly, these tests are often the same tests regularly performed by coaches and practitioners around the world. The findings from this study can contribute to the refinement of assessment protocols characteristically utilized by ice hockey practitioners. Additionally, this evidence supports coaches and trainers in focusing on dryland activities and training that enhance speed, agility, acceleration, and jumping power in the early years of ice hockey development. Important next steps to this work should examine the underlying sport-specific physiology underpinning on-ice performance; however, this study contributes a valuable extension of previous work in adult populations to better inform training protocols and foster long-term player development.

Liu et al. examined the utility of complex training as a resistance training method, combining high-load resistance training with plyometrics, the pairing of which is thought to improve strength and explosive power compared to strength or power training alone. The authors aimed to evaluate whether 8 weeks of strength training unilaterally, bilaterally or a combination of the two offered greater gains in strength and power. They demonstrated that all three strength intervention strategies equally improved upper and lower body strength and power, with only significant group differences found between peak power output at 30% of 1 repetition maximum. This may suggest that a unilateral or a combination of unilateral and bilateral strength training regimes is superior to bilateral training alone, at least for this specific measure of strength and power.

Finally, Yogev et al. reported on the effects of endurance training on muscle oxygen (de)saturation from near-infrared spectroscopy during incremental exercise tests. This may have important training implications as previous research has demonstrated that a lower desaturation coincides with increases in maximal oxygen uptake and performance in elite sprint kayakers (11). Their systematic review and meta-analysis suggest no effect of endurance training on minimum oxygen saturation during an incremental exercise test. These findings raise important questions about whether the minimum muscle oxygen desaturation is indicative of systemic cardiovascular changes or peripheral adaptations to endurance training.

## Future directions

This Research topic provides coaches and practitioners with new insights into the dose-response relationship of different forms of exercise, and the interplay between physiological function and human performance. Although exercise adaptations are often evaluated using a pre-and post-mesocycle approach, this form of assessment is retrospective in nature, and limits the understanding of the athlete's response over the duration of the training period. Unremitting developments in wearable technology allow integrative support staff to monitor the athlete's physiological response during and post-exercise (i.e., dose-response) in real time. This immediate feedback allows for evidence-based decisions to adjust present training sessions to mitigate fatigue, or include additional exercise, to ensure athletic development over the course of each mesocycle. Sports scientists working in a team environment are encouraged to publish comprehensive data sets that demonstrate the utility of wearable technology in examining the dose-response of daily training in elite athletes. Taken together, this information can be used to develop consensus statements to establish best practice guidelines for integrative support staff working within professional, national, and provincial sports organizations.
